# Cooperation of Sumoylated Chromosomal Proteins in rDNA Maintenance

**DOI:** 10.1371/journal.pgen.1000215

**Published:** 2008-10-10

**Authors:** Yoshimitsu Takahashi, Stanimir Dulev, Xianpeng Liu, Natalie Jasmin Hiller, Xiaolan Zhao, Alexander Strunnikov

**Affiliations:** 1 Laboratory of Gene Regulation and Development, National Institute of Child Health and Human Development, National Institutes of Health, Bethesda, Maryland, United States of America; 2 University of Plovdiv, Plovdiv, Bulgaria; 3 Molecular Biology Department, Memorial Sloan Kettering Institute, New York, New York, United States of America; 4 The Rockefeller University, New York, New York, United States of America; Yale University, United States of America

## Abstract

SUMO is a posttranslational modifier that can modulate protein activities, interactions, and localizations. As the GFP-Smt3p fusion protein has a preference for subnucleolar localization, especially when deconjugation is impaired, the nucleolar role of SUMO can be the key to its biological functions. Using conditional triple SUMO E3 mutants, we show that defects in sumoylation impair rDNA maintenance, i.e., the rDNA segregation is defective and the rDNA copy number decreases in these mutants. Upon characterization of sumoylated proteins involved in rDNA maintenance, we established that Top1p and Top2p, which are sumoylated by Siz1p/Siz2p, most likely collaborate with substrates of Mms21p to maintain rDNA integrity. Cohesin and condensin subunits, which both play important roles in rDNA stability and structures, are potential substrates of Mms21, as their sumoylation depends on Mms21p, but not Siz1p and Siz2p. In addition, binding of cohesin and condensin to rDNA is altered in the *mms21-CH* E3-deficient mutant.

## Author Summary

Disruption of the SUMO (small ubiquitin-like modifier) pathway by mutations is lethal in mammals and in budding yeast; however, the essential nature of its role remains unknown, mainly because only a small fraction of most substrate proteins is SUMO-modified. We argue that the clustering of SUMO modifications among subunits of multiprotein complexes or within biochemical pathways indicates that SUMO-modified fractions of target proteins may have specific cooperative activities, distinct from the functions of individual unmodified proteins. SUMO conjugation-mediated functions in nucleolar processes can potentially be examples of such specific cooperative pathways, as we show that SUMO conjugates have a strong preference for nucleolar localization in budding yeast. Moreover, we demonstrate that stable maintenance of the nucleolar DNA and nucleolus is dependent on the putative functional interaction between the sumoylation of topoisomerases I and II (by Siz1p/Siz2p) and substrates of Mms21p SUMO-conjugating activity.

## Introduction

SUMO covalently modifies substrate proteins and can modulate their activities [1],[2]. Similar to the ubiquitination pathway, sumoylation requires E1, E2, and E3 steps to conjugate SUMO to substrates [1],[2]. In budding yeast, genes encoding SUMO (*SMT3*) as well as SUMO E1 (*UBA2*, *AOS1*) and E2 (*UBC9*) are essential [3]–[7], indicating that sumoylation regulates key processes in cell survival. Indeed, recent studies have shown that sumoylation participates in multiple cellular pathways, many of which are intranuclear, such as transcription, DNA repair, and nuclear domain organization [1],[2],[8].

Despite the importance of sumoylation in cell physiology, only a handful of substrates have been studied in detail, partly due to the low abundance of sumoylated fractions of a given protein [9]–[11]. Nevertheless, recent studies have revealed several salient features of sumoylation. Proteomic research showed that multiple proteins in the same protein complexes or biochemical pathways are sumoylated; such “clustered” SUMO modifications raise the possibility that sumoylated versions of cellular proteins may cooperate for specific functions [12]–[17]. This could provide an explanation for the low level of modification for the majority of SUMO substrate proteins. The putative cooperation may also be reflected in specific subnuclear localization of sumoylated proteins [11], [18]–[21]. The recent characterization of SUMO-binding domains within proteins localized to nuclear subdomains suggests that SUMO modification is recognized by receptor-like proteins, thus providing a mechanism for subnuclear domain organization by SUMO [22]–[26]. This targeting role of SUMO, and its potential ensuing ability to establish a novel set of protein-protein contacts, may be key to its essential biological roles.

In *S. cerevisiae*, one prominent subnuclear domain relevant to SUMO biology is the nucleolus, as our previous results showed that the green fluorescent protein (GFP) fusion to Smt3p has a preference for nucleolar localization when deconjugation is impaired [11]. In the present study, we examine the potential cooperation of several chromosomal proteins known to be involved in the stability of ribosomal RNA genes (rDNA). Among these proteins are two topoisomerases, Top1 and Top2, which facilitate rDNA transcription and replication. Top1 and Top2 are sumoylated by two paralogous SUMO E3s, Siz1 and Siz2, which are responsible for the majority of sumoylation in yeast. The third SUMO E3, Mms21, is a subunit of the Smc5/Smc6 complex, which also binds to rDNA and maintains its stability [27]–[29]. The relevant substrates of Mms21p E3 activity in rDNA maintenance are not known. We examined conditional triple mutants lacking E3 activities of Siz1, Siz2, and Mms21 and found that rDNA stability is severely impaired in these mutants. Furthermore, the Mms21p E3 activity is essential in the absence of Top1p and sumoylated Top2p. Thus, our results show that Top1p and Top2p most likely cooperate in the rDNA maintenance pathway with Mms21p substrates, including the Structural Maintenance of Chromosomes (SMC) complexes cohesin and condensin.

## Results

### SUMO Is Prominently Enriched in the Nucleolus

**Figure 1:**
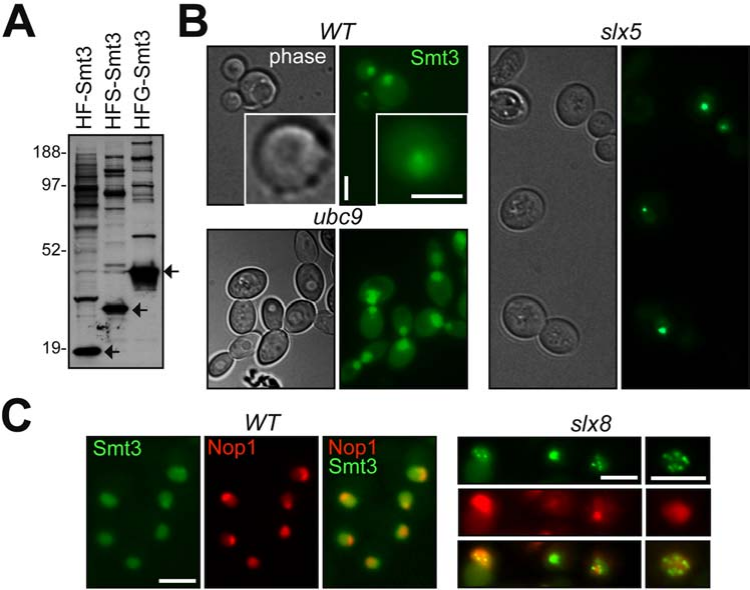
Smt3p conjugates are enriched in the nucleolus. (A) Alternative tagging of Smt3p expressed at the native levels enables the identification of sumoylated proteins. Total sumoylated proteins were purified by IMAC from the strains expressing wild-type levels of poly-His/FLAG-tagged Smt3p (HF-Smt3, 924-YPH499b), poly-His/FLAG/S-tag-Smt3p (HFS-Smt3, 1008-YPH499), and poly-His/FLAG/GFP-Smt3p (HFG-Smt3, 1014-YPH499. Sumoylated proteins here and thereafter are separated by PAGE and detected by Western-blotting using the anti-FLAG antibody. Arrows indicate the proportional size shifts between the free SUMO forms. Molecular weight markers (×1000) are shown on the left. (B) GFP-SUMO localization as a function of conjugation/de-conjugation. The wild type (1014-YPH499b), *ubc9-1* (1cYT630), and *slx5Δ* (1dYT631) strains expressing GFP-Smt3p as in (A) were incubated at 32°C (semi-permissive for *ubc9-1*) for 5 h and imaged live. The insert shows gradient-like distribution of SUMO typical for wild type, which is noticeable at higher magnifications. Scale bars here and elsewhere are 5 µm. (C) SUMO conjugates are concentrated in the nucleolus in *slx8Δ* cells. The wild type (1014-YPH499) and *slx8Δ* (1aYT629) strains co-expressing GFP-Smt3p and Nop1p-mRFP were incubated at 30°C; cell images were captured live.

We recently showed that the bulk of sumoylated proteins are concentrated in a subnucleolar area reminiscent of rDNA chromatin, if the nuclear desumoylation enzyme Smt4p is inactivated by the *smt4* gene deletion [11]. This observation suggested that the SUMO pathway may play a major role in the nucleolus. Therefore, we closely examined strains expressing GFP-Smt3p under the native *SMT3* promoter, as a sole source of SUMO. To monitor the GFP-Smt3p (HFG-Smt3) modification biochemically we also generated a shorter fusion (with the extended S-tag, HFS-Smt3). Similar patterns of conjugated protein bands were observed for both strains, except bands shifted accordingly to the tag size (Figure 1A). Both the HFS-Smt3 and HFG-Smt3 strains had the wild type doubling time (not shown), indicating that HFS-Smt3p and HFG-Smt3p fulfill key functions of SUMO.

In wild type cells, the GFP-Smt3p signal was nuclear with a gradient-like appearance (Figure 1B, insert). This subnuclear pattern of localization depends on SUMO conjugation, as GFP-Smt3p was uniformly filling the whole nucleus in *ubc9* mutants (Figure 1B). In contrast, in *slx5Δ* or *slx8Δ* mutants, which accumulate poly-sumoylated targets, similarly to *smt4Δ* strains [23]–[25], GFP-Smt3p was concentrated in several foci located in the nucleolus (Figure 1B, 1C). These results suggest that in wild type a large portion of sumoylated nuclear proteins is localized in the nucleolus, at least transiently.

### Triple SUMO E3 Mutants Are Defective in rDNA Segregation and Maintenance

**Figure 2:**
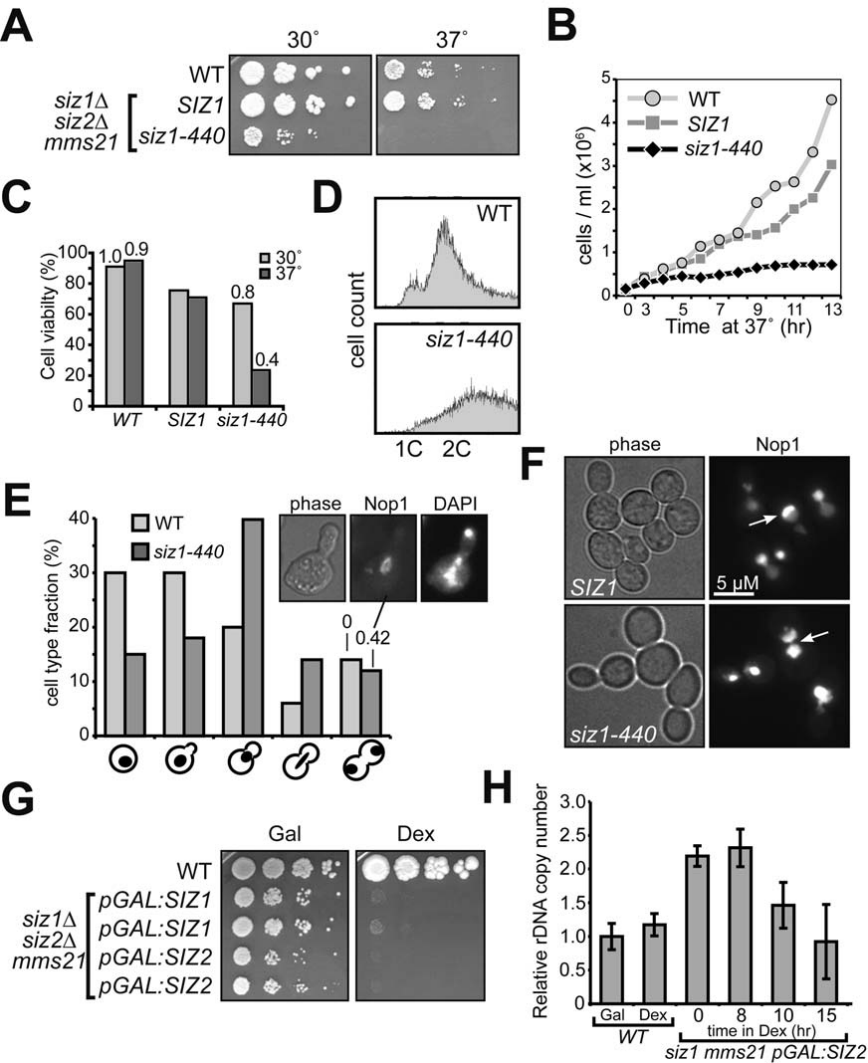
Conditional E3 mutants exhibit defects in rDNA segregation and maintenance. (A) Characterization of a conditional SUMO E3 mutant (E3-ts). The conditional E3 strain (1YT632, *siz1Δ*, *siz2Δ mms21-CH*) contains *siz1-440* encoding a truncated Siz1p that lacks the COOH-terminal region. The control strains shown are wild type (*WT*, W303-1A) and a *siz1Δ siz2Δ mms21-CH* strain (1cYT628) bearing a full-length *SIZ1* plasmid. 10-fold serial culture dilutions were spotted on YPD plates and incubated for 2 days at indicated temperatures. (B) E3-ts cells arrest within two cell divisions at the restrictive temperature. The strains as in (A) were grown exponentially in YPD at 30°C and shifted to 37°C at zero timepoint. Cell aliquots were removed at each subsequent time point and cells were counted using a haemocytometer. (C) E3-ts cells lose viability and rDNA copies at non-permissive temperature. The three strains as in (A) were grown in YPD at 30°C or shifted to 37°C for 5 hours to determine cell viability by plating assay. The relative rDNA copy number (shown adjacent to viability bars) was determined for wild type and the *siz2Δ mms21-CH siz1-440* strain as in [11] and normalized to wild type value at 30°C. (D) The majority of E3-ts mutant cells have replicated DNA. Exponentially growing cells (as in A) were shifted to 37°C for 5 hours, and DNA content was analyzed by FACS. (E) The nucleolar segregation defect in E3-ts cells at a non-permissive temperature. Haploid E3-ts cells were incubated at 37°C for 5 hours, stained with DAPI and examined microscopically. Strains were as in 1A, except they contained the nucleolar marker Nop1p-mRFP. The numbers above the bars indicate the fraction of anaphase cells, which have segregated DAPI signals but unsegregated nucleoli (the micrograph shows an example). (F) E3-deficient cells have altered nucleolar morphology. The *SIZ1* (NOP1-1cYT628) and *siz1-440* (NOP1-1YT632) strains were treated as in (E). While all *SIZ1* cells have proper nucleolar morphology (Nop1-mRFP marker), nucleolar material is more dispersed in E3-ts cells. Similar results were obtained using another nucleolar marker Sik1p-mRFP (not shown). (G) *SIZ1* or *SIZ2* transcription shut-off in the *siz1Δ siz2Δ mms21-CH* strain leads to growth arrest. Cultures of triple-mutant (*siz1Δ siz2Δ mms21-CH*) strains carrying either *pGAL*∶*SIZ1* (10aYT633) or *pGAL*∶*SIZ2* (11aYT634) were plated on media containing either galactose (*Gal*) or glucose (*Dex*), and incubated for 3 days at 30°C. (H) The levels of SUMO E3 activity affect rDNA stability. Cultures of wild type or triple-mutant strain (*siz1Δ siz2Δ mms21-CH*) carrying *pGAL*∶*SIZ2* (11aYT634) were processed for rDNA copy number analysis as in [11] after a shift from galactose media to dextrose. The *pGAL*∶*SIZ2* cells break through the metaphase arrest after about 9 h in glucose (data not shown).

All three known *S. cerevisiae* SUMO E3 activities have been implicated in the regulation of rDNA maintenance and nucleolar stability: *siz1Δ siz2Δ* strains exhibit 60% of wild-type levels of the rDNA copy number [11], while the *mms21-11* mutation leads to fragmentation of the nucleolus [28]. In order to elucidate the role of SUMO E3s in nucleolar maintenance, we examined strains conditionally lacking E3 activity, because the combination of *siz1Δ siz2Δ* and the *mms21-CH* mutation (i.e. mutated cysteine and histidine in the SP-RING-like domain of Mms21p) was lethal (not shown). To this end, we introduced the *siz1Δ440* allele [9] into the *siz1Δ siz2Δ mms21-CH* triple mutant. The protein encoded by *siz1Δ440* retains the SP-RING domain and E3 activity *in vitro* but lacks the regulatory domain and fails to localize properly *in vivo* [9]. This mislocalization may be the reason for the temperature sensitivity of the *siz1Δ440 siz2Δ mms21-CH* strain (called E3-ts thereafter; Figure 2A).

The E3-ts strain stopped growing after one or two divisions at 37°C (Figure 2B, 2C). It exhibited a 2.5-fold decrease in rDNA content (as determined by quantitative polymerase chain reaction, qPCR, Figure 2C) while the bulk of DNA replication was completed (Figure 2D). The decrease of the rDNA copy number in E3s-ts was more severe than in *siz1Δ siz2Δ* or *mms21-CH* strains [11] (and Figure 2C), suggesting that all three E3s are required to maintain rDNA stability. Furthermore, 42% of the E3s-ts cells with separated nuclei had unsegregated rDNA (Figure 2E); and most cells' nucleoli were highly variable in size and did not acquire the characteristic crescent shape (Figure 2F). These results show that lacking all three SUMO E3 activities dramatically affects nucleolar stability.

To verify this conclusion, we analyzed E3-shut-off strains expressing Siz2p or Siz1p under the *GAL*-promoter in *siz1Δ siz2Δ mms21-CH* cells. As expected, such strains could not grow on a medium containing glucose (Figure 2G). A correlation of SUMO E3 levels and rDNA copy number was found upon analysis of these strains: constant *SIZ2* overexpression induced doubling of the rDNA copy number, while *SIZ2* shut-off resulted in an efficient loss of extra rDNA (Figure 2H). This loss was likely due to missegregation of rDNA, as it coincided with cell division after a prolonged arrest in dextrose (around 9 h). Thus, the results obtained with both types of conditional E3 mutants suggest that SUMO conjugations mediated by Siz1p, Siz2p, and Mms21p are important for proper rDNA segregation and/or copy number control.

### Genetic Interactions between *mms21-CH*, *top1*, and *top2* Mutations Suggests a Common Pathway in rDNA Maintenance

**Figure 3:**
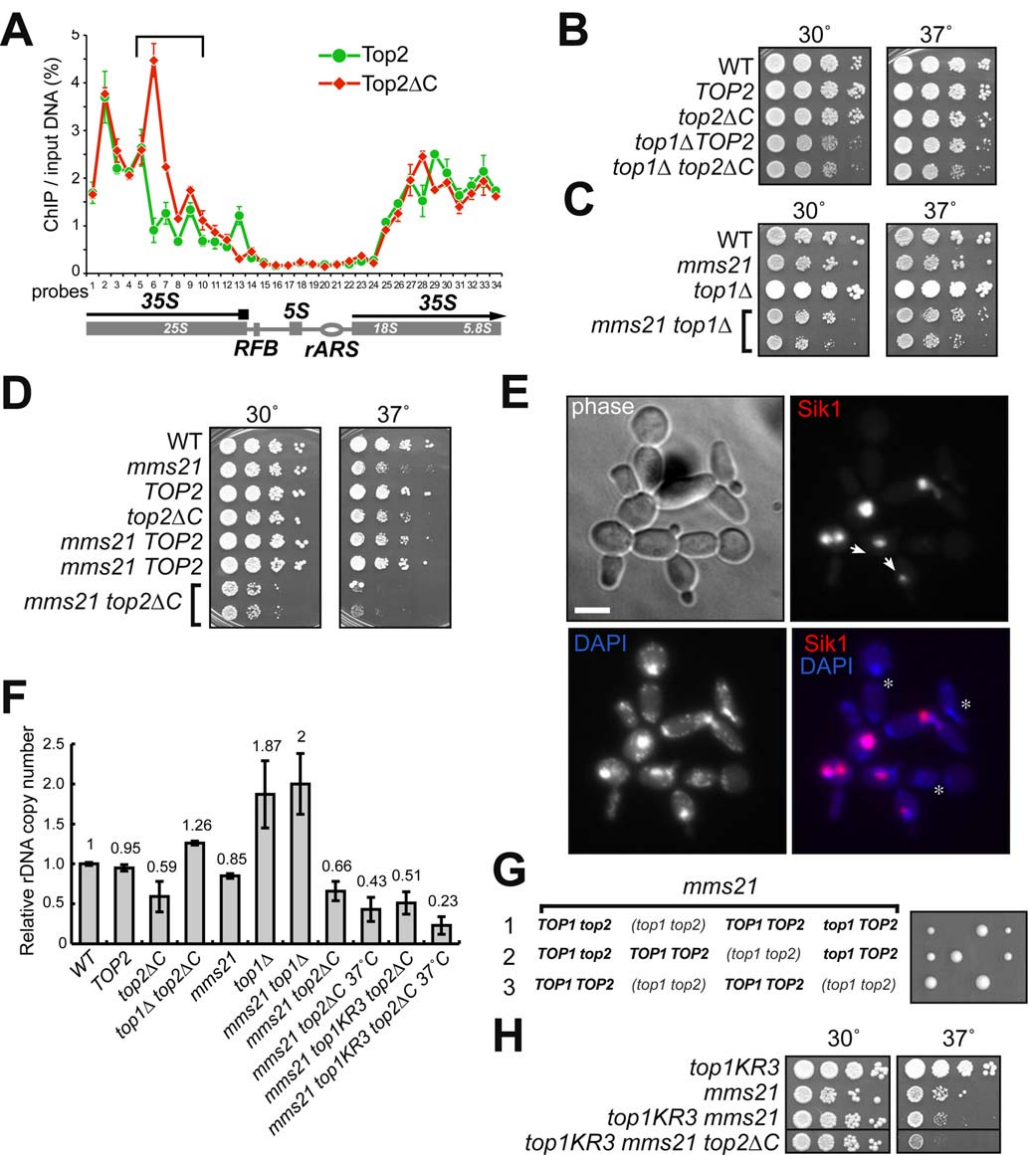
*mms21-CH* exhibits genetic interactions with *top1* and *top2* mutations affecting topoisomerase sumoylation. (A) The rDNA binding pattern of Top2pΔC differs from that of Top2p. Wild type *TOP2* (1033-W303) and *top2ΔC* (1035-W303) strains, both alleles HA-tagged, were analyzed by ChIP/qPCR using the probes covering the whole rDNA repeat as described in [45]. The cells were arrested by nocodazole for 3 hr at 30°C prior to chromatin cross-linking and extraction. (B) Genetic interactions of *top1Δ* and *top2ΔC*. Wild type strain (*WT*, W303-1A) was compared for growth fitness with the following strains: HA-tagged *TOP2* (1033-W303), *top2ΔC* (1035-W303), *top1Δ TOP2* (1aYT624), and *top1Δ top2ΔC* (1aYT625). Incubation was for 2 days. (C) Genetic interaction between *top1Δ* and *mms21-CH*. Experimental conditions were as in (B); wild type, *top1Δ*, *mms21-CH* (17-YT635) and *top1Δ mms21-CH* (22aYT636) strains were compared. The small colony size was noticeable for the double mutant at both 30°C and 37°C. (D) Genetic interaction between *top2ΔC* and *mms21-CH*. The *mms21-CH* (17-YT635), wild type (W303), *TOP2* (tagged copy, 1033-W303), *top2ΔC* (1035-W303), *mms21-CH TOP2* (15cYT636), and *mms21-CH top2ΔC* (16cYT637) strains were incubated for 2 days. The *mms21-CH top2ΔC* double mutant had tight growth arrest at 37°C (colonies growing at 37°C were *top2ΔC* excision revertants). (E) Nucleolar defects in the *top2ΔC mms21-CH* mutant. *mms21-CH top2ΔC* (16cYT637) cells expressing a nucleolar marker (Sik1p-mRFP) were shifted to 37°C and stained with DAPI. More than 95% of cells were inviable after 4 hours at 37°C, and a high proportion (up to 40%) of cells had no nucleoli (asterisks). In some cases of delayed cytokinesis, the putative sequence of nucleolar missegregation and diminution could be traced (arrows). (F) Relative rDNA copy number in *mms21-CH*, topoisomerase mutants and combination mutants. The rDNA copy number was determined by qPCR as in [11] and normalized to the wild-type levels. At least four independent clones were analyzed for each genotype. Strains are as in (B), (C), (D), and (H). (G) *top1Δ top2ΔC mms21-CH* triple mutant is inviable. Three representative tetrads incubated at 30°C for 3 days are shown, where spores were allowed to germinate after dissection of the diploid (YT638) homozygous for *mms21-CH* and heterozygous for both *top1Δ* and *top2ΔC*. More than 30 tetrads were dissected. Inviable spores were *top1Δ top2ΔC mms21-CH* triple mutants, as was deduced from the genotypes of sibling spore clones. (H) Cells with reduced Top1p sumoylation require both Top2p sumoylation and Mms21 E3 activity for optimal growth. *top1KR3* (EJY457), *mms21-CH* (17-YT635), *top1KR3 mms21-CH* (21cYT639), and *top1KR3 mms21-CH top2ΔC* (22cYT640) strains were analyzed as in (B). The corresponding relative rDNA copy number is shown in (F).

Due to the large number of sumoylated nucleolar proteins [30],[31], it is not feasible to analyze all of them simultaneously. However, it is known that, among SUMO substrates, Top1 and Top2 topoisomerases are involved in the maintenance of tandem rDNA array stability [32]. Moreover, we have previously shown that *top2ΔC* and *top2-SNM* alleles, with either complete or partial loss of *in vivo* Top2p sumoylation, respectively [20],[33] (and unpublished data), result in a decrease of the rDNA copy number [11]. Consistent with the role of Top2 sumoylation in rDNA regulation, Top2ΔC ChIP from nocodazole-arrested cells (sumoylation levels of Top2p are maximal in mitosis [33]) showed a strong peak towards the end of the 35S RNA gene, which was not present in wild type (Figure 3A). The specific location of this enrichment may indicate possible impediments to Pol I transcription and/or DNA replication, which are characteristic of more penetrant *top2* mutants [34].

While the Figure 3A results, as well as previous work [11], suggest that sumoylation levels affect both the localization and function of Top2p in rDNA, the healthy growth rate of the *top2ΔC* strain indicates the existence of some redundant activities. One candidate is Top1p, which is able to alleviate DNA replication and transcription constraints induced by *top2* mutants [34]–[37]. The other candidate is Mms21p; as its SUMO E3 activity may play a role in counteracting replication stress upon DNA damage [29],[38]. To investigate whether sumoylation of Top2p has any redundancy in rDNA maintenance with either Top1p or sumoylation mediated by Mms21p, we combined *top2ΔC*, *top1Δ*, and *mms21-CH* mutations in pairs and examined cell growth and rDNA levels in each double mutant. In all cases we uncovered some synthetic phenotype. The interaction was relatively mild between *top1Δ* and either *top2ΔC* (Figure 3B) or *mms21-CH* (Figure 3C), but strong for *top2ΔC* and *mms21-CH*, resulting in a tight ts-phenotype in this double mutant (Figure 3D, only revertants grow at 37°C). With respect to rDNA copy number, the *mms21-CH* mutation was epistatic to either *top1Δ* or *top2ΔC* mutations (Figure 3F), however the *top2ΔC mms21-CH* strain showed ample signs of nondisjunction and/or loss of nucleolar material in cell divisions at a nonpermissive temperature (Figure 3E, 3F). In contrast, chromosome III loss was not increased in the *top2ΔC mms21-CH* diploid (data not shown). These results indicate that some targets of Mms21p sumoylation may have rDNA activities redundant with sumoylated Top2p.

The fact that at least some growth inhibition was observed in cells with all double combinations of *top1Δ*, *top2ΔC* and *mms21-CH* mutations (Figure 3B, 3C, 3D) may indicate some functional redundancy between the three corresponding activities disrupted by these mutations. To test this, we constructed the triple *top1Δ top2ΔC mms21-CH* mutant and found that such a combination was lethal even at permissive temperatures (Figure 3G). Next, we constructed the triple *top1Δ top2-SNM mms21-CH* mutant, which has only trace sumoylation of Top2p *in vivo* (in contrast to a complete lack of sumoylation in Top2*ΔC*) [20] (and unpublished results), and showed that it had a severe inhibition of growth (Figure S1). These two results (Figure 3G and Figure S1) are consistent with the hypothesis that Top1p activity becomes essential when both the Mms21 E3 activity and Top2p sumoylation are blocked. To understand whether such a function of Top1p is itself related to Top1 sumoylation, we examined the genetic interactions of *top1KR3*, which lacks three major Top1p sumoylation sites [39], with *mms21-CH* and *top2ΔC*. The triple *top1KR3 top2ΔC mms21-CH* mutant had a severe synthetic growth phenotype (Figure 3H) as well as prominent rDNA loss (Figure 3F). This result indicates that Top1p activity in rDNA could be also SUMO-dependent. It also complements the conclusions based on Figure 3G and Figure S1, suggesting that Top1p, sumoylated targets of Mms21p, and sumoylated Top2p may act together in rDNA maintenance.

### Condensin and Cohesin Are Directly Regulated by Mms21-Mediated Sumoylation

**Figure 4:**
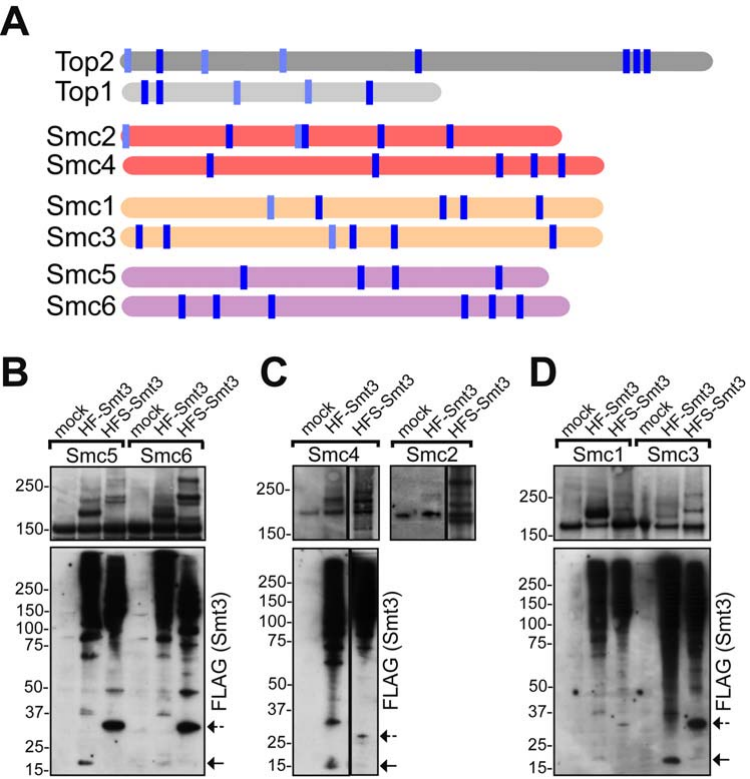
SMC proteins are sumoylated. (A) Predicted sumoylation sites in topoisomerases and SMC proteins. Sumoylation sites were predicted with the SUMOplot™ algorithm (Abgent). Vertical blue lines indicate the positions of potential sumoylation sites: dark - a high score for predicted sites (>0.9), light - lower scores (0.8–0.9). (B–D) SUMO modifications of the six SMC protein complexes are revealed by differential Smt3p tagging. Each of the SMC proteins was tagged with 5xHA; strains also contained differentially tagged Smt3p as indicated. Cells were treated with nocodazole prior to conjugate purification by IMAC. The arrows indicate un-conjugated forms of tagged SUMO. Sumoylated SMC proteins were detected according to the principle in Figure 1A by anti-HA antibody, except for Smc2p (specific anti-Smc2 antibody).

While the specific targets of Mms21 E3 activity are not well characterized, especially in the unchallenged (i.e. no DNA damage) cell cycle, one can hypothesize (based on the Figure 3 results) that these substrates include proteins complementing topoisomerase activities in the nucleolar organizer. SMC complexes are obvious candidates: they change the topological state of DNA [40], the Smc5p/Smc6p complex and condensin are essential for rDNA maintenance and segregation [27],[41], and cohesin is apparently involved in the rDNA amplification pathway [42]. Incidentally, some subunits of the three SMC complexes can be sumoylated [12],[13],[15], although only Smc5p sumoylation is partially dependent on Mms21p [28]. Furthermore, all SMC proteins contain multiple potential sumoylation sites (Figure 4A).

These considerations prompted us to examine whether the sumoylation of SMC proteins contributes to rDNA maintenance. First, we assayed the comparative extent of SMC proteins' sumoylation. Using the alternative tagging of Smt3p with variable-length tags (HF and HFS, Figure 1A), we investigated the sumoylation levels of all six SMC proteins and showed that they were SUMO-modified in mitosis (Figure 4 B, C, D). The HFS versus HF tag super-shifts of gel-retarded SMC bands established their identity as products of sumoylation; therefore we limited the subsequent analysis to the HF-tagged Smt3p only. In most cases, more than two sumoylated bands were evident, indicating that SMC proteins were SUMO-modified at multiple sites or they were polysumoylated (Figure 4 B, C, D). In the case of condensin, which is known to interact with topoisomerases functionally [43],[44], but showed the weakest sumoylation (Figure 4C), we detected reproducible SUMO modifications of non-SMC subunits as well (Figure S2).

**Figure 5:**
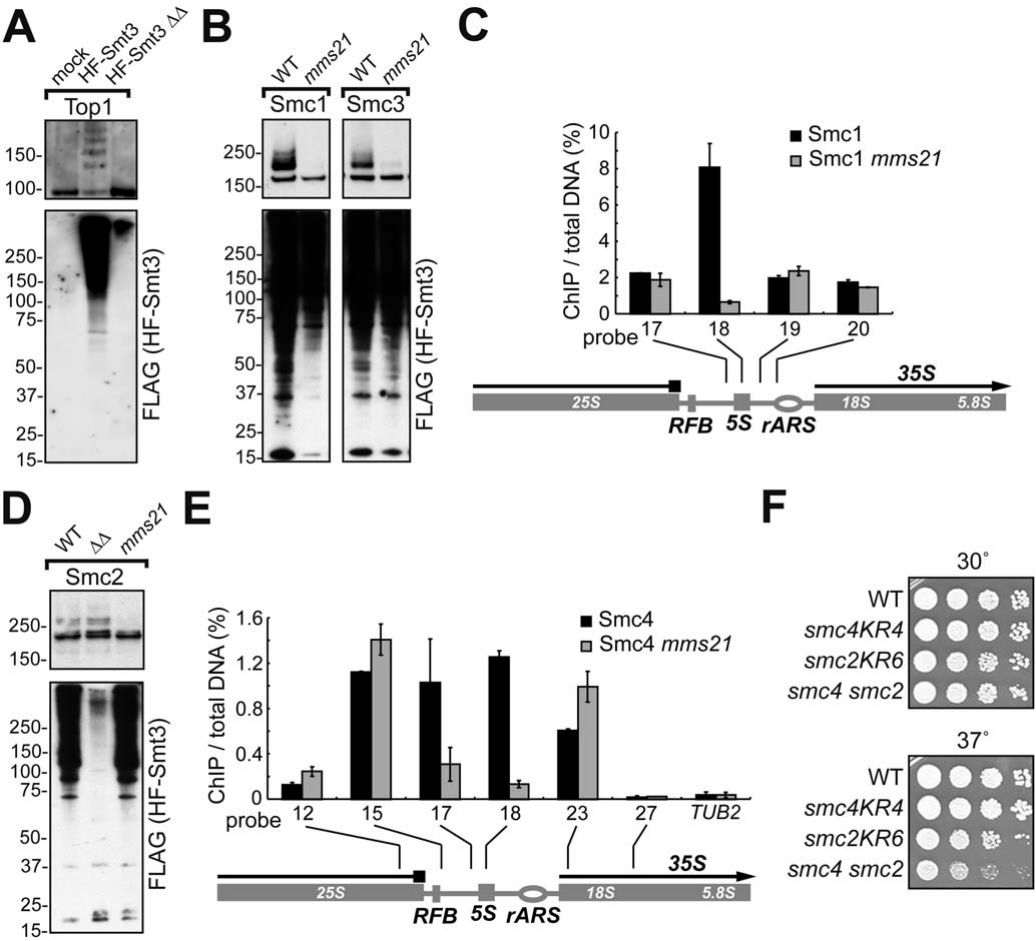
*mms21-CH* affects sumoylation and rDNA binding of cohesin and condensin. (A) Top1p sumoylation requires Siz1p/Siz2p. SUMO conjugates were purified from Siz^+^ (924-1111-W303) or *siz1Δ siz2Δ* (*ΔΔ*,1aYT641) strains with tagged Top1p (*TOP1*∶5HA) and SUMO (*HF-SMT3*). Top1p was detected by the anti-HA (16B12) antibody. (B) Sumoylation of Smc1p and Smc3p is dependent on Mms21p. SUMO conjugates were purified from wild-type *MMS21* (*SMC1*: 924-1131-W303, *SMC3*: 924-1133-W303) or *mms21-CH* (*SMC1*: 1YT645, *SMC3*: 1YT644) cells. Smc1p and Smc3p were detected by anti-HA antibody. (C) Smc1p lacking sumoylation exhibits altered rDNA binding. Wild type (924-1131-W303) and *mms12-CH* (1YT645) strains, both carrying HA-tagged Smc1p, were arrested by nococodazole and analyzed by ChIP/qPCR using the set of probes covering the whole rDNA repeat [45]. Only the probes relevant to the known cohesin enrichment peak [42],[61] and negative controls are shown. (D) Sumoylation of Smc2p is dependent on Mms21p but not on Siz1p/Siz2p. SUMO conjugates were purified from wild type, *siz1Δ siz2Δ* (*ΔΔ*, 1bYT642) and *mms21-CH* (1cYT643). Smc2p was detected by the specific anti-Smc2p antibody [41]. (E) Smc4p has reduced enrichment at the 5S rRNA gene in *mms12-CH*. The wild type (924-1134-W303) and *mms12-CH* (1YT646) strains, both carrying HA-tagged Smc4p, were arrested by nocodazole for 3 hr at 30°C and analyzed by ChIP/qPCR using the set of probes covering the whole rDNA repeat as described in [45]. Only the probes relevant to the known condensin enrichment peaks [45] and negative controls are shown. (F) Mutations of potential sumoylation sites of Smc2p and Smc4p lead to synergistic growth defects. Wild type (W303), *smc2KR6* (1146-YT656), *smc4KR4* (1YT657), and double-mutant *smc2KR6 smc4KR4* (1YT658) strains were tested for growth at 30°C and 37°C.

Next, we examined whether sumoylation of SMC proteins in nocodazole-arrested cells depends on Mms21p or Siz1p/Siz2p. We found that, while Top1p sumoylation was wholly dependent on Siz1p/Siz2p (Figure 5A), Mms21p was responsible for sumoylation of Smc1p, Smc3p (Figure 5B), Smc2p (Figure 5D), and partially of Smc4p and Ycs4p (data not shown). Concurrently, *siz1Δ siz2Δ* double mutants had no effect on sumoylation of Smc2p (Figure 5D), Smc1p and Smc3p (data not shown). Sumoylation of Smc5p was a combination of Siz1p/Siz2p and Mms21p activities; and the Smc6p sumoylation was largely dependent on Siz1p/Siz2p (data to be published elsewhere). These results show that mitotic SUMO-modifications of cohesin and condensin are largely dependent on Mms21p. In the experiments *in vitro*, recombinant Mms21p failed to sumoylate either condensin or cohesin bound to chromatin (data not shown), indicating that the Smc5/Smc6 holocomplex is most likely required for Mms21 SUMO E3 activity, which is consistent with the stoichiometric binding of Mms21p to the Smc5/Smc6 complex *in vivo* [28],[29].

The above results suggest that sumoylation of cohesin and condensin (among other Mms21p substrates) could possibly mediate some of the rDNA-specific functions conferred by the Mms21p E3 activity. If this is the case, the *mms21-CH* mutation may have an effect on the distribution of cohesin and condensin in rDNA. Therefore, we tested the enrichment of cohesin and condensin at rDNA in *mms21-CH* cells by ChIP. In wild type, both Smc1p and Smc4p had well-defined peaks of rDNA enrichment (Figure 5C, 5E), as was expected [41],[45],[46]. In *mms21-CH* cells, condensin retained proper enrichment at its RFB and the 35S RNA gene (5′) peaks, both of which are important for rDNA condensation and segregation [45], [47]–[49] (Figure 5E). However, the 5S rRNA gene-neighboring peaks of both cohesin (Figure 5C) and condensin (Figure 5E) were eliminated, suggesting that Mms21p E3 activity controls the binding of both complexes to the 5S rRNA gene.

### Condensin Sumoylation Is Essential in the Absence of Top1p and Sumoylated Top2p

As the role of cohesin in rDNA appears to be nonessential [42], we turned our attention to condensin sumoylation. To further understand the function of sumoylation of condensin subunits we generated a set of mutations replacing six putative acceptor lysine residues with arginine in Smc2p and four in Smc4p (*smc2KR6* and *smc4KR4* alleles). Both alleles displayed reduced sumoylation (Figure S3), however neither had growth defects at 30°C or 37°C. Nevertheless, the combination of the two yielded temperature sensitivity (Figure 5F). We then attempted to recapitulate the lethality of the *top1Δ top2ΔC mms21-CH* triple mutant (Figure 3G) with *top1Δ*, *top2ΔC*, and *smc2KR6/smc4KR4* alleles. Tetrad analysis of diploid strains (YT660) homozygous for *top1Δ* and heterozygous for both *smc2KR6* and *top2ΔC* showed that the triple combination of *smc2KR6*, *top1Δ*, and *top2ΔC* was already lethal: no *top2ΔC* alleles were found in 120 examined *smc2KR6/top1Δ* spores. Thus, condensin sumoylation apparently acts in a pathway that is genetically redundant (and possibly molecularly cooperative in wild type) with Top1p and SUMO-dependent Top2p activities. However, one caveat of this interpretation is that we cannot exclude the possibility that *smc2KR6*, as a result of multiple lysine residue substitutions, may affect other aspects of Smc2p function besides sumoylation.

## Discussion

While temperature-sensitive alleles of the SUMO pathway genes (*ubc9-1*, *ulp1-333*, *smt3-331*) show severe cell cycle defects, the requirement of SUMO for cell viability in budding yeast is largely unexplained. However, the persistently low levels of target modifications by SUMO is a likely indicator that sumoylated fractions of proteins have biological roles that are distinct from the non-sumoylated pools of the same proteins. Considering the enrichment of sumoylated proteins at some nuclear subdomains, it is plausible that the essential roles of SUMO include specific activities of sumoylated proteins at these subnuclear regions, such as the centromere [20],[50] and the nucleolus [11]. In our previous and current work we showed that the SUMO conjugates became predominantly nucleolar when the removal of conjugates was compromised (Figure 1B, 1C and [11]). Furthermore, the population of cells that breaks through the metaphase arrest induced by SUMO E3 dysfunction is prone to nucleolar nondisjunction (Figure 2E), which might account for the rDNA loss in these triple E3-deficient cells (Figure 2C, 2H). Thus, nucleolar proteins, particularly ones functioning in rDNA chromatin, can be key targets of SUMO in the nucleus.

What essential rDNA functions might require SUMO? Three key factors are known to contribute to the stability of rDNA arrays: (1) silencing, i.e. suppression of sister chromatid recombination and ensuing formation of extrachromosomal rDNA [51],[52]; (2) amplification controls maintaining the optimal size of the rDNA array [42],[51]; and (3) proper rDNA segregation [27], [45], [53]–[55]. Topoisomerases probably participate in all of these processes and, with respect to SUMO, our previous work suggests that lacking Top2p sumoylation can lead to rDNA defects, as the rDNA array stability was reduced to a similar degree in *top2ΔC*, *top2-SNM*, or *siz1Δsiz2Δ* cells [11]. Thus, we proposed a hypothesis [11] that the *top1Δ top2ΔC* double mutant essentially phenocopies the rDNA destabilization phenotype of more severe *top1Δ top2* double mutants [32].

In the present work we made the first step in testing the more general idea that other sumoylation targets cooperate with sumoylated Top2p in maintaining rDNA stability. In the process we uncovered an important pathway controlled by Mms21p in the unchallenged cell cycle. Indeed, analysis of the *mms21-CH top2ΔC* double mutant showed that the combined deficiency in Top2p sumoylation and in Mms21p SUMO E3 function do lead to strong rDNA segregation defects (Figure 3E). These defects are exacerbated by the loss of Top1p activity or Top1p sumoylation in corresponding triple mutants (Figure 3F, 3G. 3H). While at this junction we cannot attribute the lethality of *top1Δ top2ΔC mms21-CH* either exclusively to the loss of sumoylation or solely to rDNA dysfunction, it does raise the possibility that Siz1/Siz2-sumoylated Top1p and Top2p cooperate with Mms21-sumoylated proteins in rDNA maintenance. Additionally considering that the mitotic sumoylation of cohesin and condensin largely depends on Mms21p (Figure 5), and that there is a synthetic lethality between *top1Δ*, *top2ΔC* and *smc2KR6* mutations, one can hypothesize that activities of sumoylated pools of condensin and cohesin are either redundant or cooperative with topoisomerases in rDNA.

More extensive experiments on the individual Mms21p substrates are needed to elucidate their molecular roles in this pathway. Top1p and Top2p are needed for both efficient transcription and DNA replication, and the Mms21p E3 activity is important to confront DNA replication-induced stress [29],[36],[38]; therefore it is possible that due to strong rDNA transcription and its asymmetric replication the resolution of transcription and replication impediments in *top1Δ top2ΔC* cells specifically requires Mms21p SUMO E3 activity. Although it remains to be tested at the molecular level, this idea is consistent with the fact that Top2p and Top1p co-localize with the Smc5p/Smc6p complex and replication/transcription landmarks genome-wide [36],[56], particularly at rDNA (S. D., to be published elsewhere).

The putative cooperation between distinct sumoylation targets (being either different proteins or different E3-specific sumoylation sites) of Siz1p, Siz2p, and Mms21p in the nucleolus may extend beyond rDNA itself. For example, many proteins involved in ribosome biogenesis are found to be sumoylated [30]; such “clustered” SUMO conjugations may be important for nucleolar integrity as well. In addition, we previously reported that condensin is enriched at the tRNA genes [57], which are transcribed by RNA Pol III. The Mms21-regulation of cohesin's and condensin's binding (Figure 5) to the 5S rRNA gene (also transcribed by Pol III) may point to a novel SUMO-controlled function common to Pol III genes, such as their recruitment to the nucleolus [58]. Further work is needed to examine these possibilities.

## Materials and Methods

Strains and plasmids used in this study are listed in Tables 1 and 2. The *E. coli* strains TOP10 and BL21(DE3) were used for cloning and protein purification, respectively. Culturing of yeast cells, microscopy, and biochemical techniques were essentially as described before [20]. *Saccharomyces cerevisiae* strains were either isogenic to W303-1A or to YPH499 (S288c) as indicated in Table 2.

**Table 1:** Plasmids.

Name	Backbone	Insert (targeting site)	Makers	Source
pAS924	pRS316	HF-*SMT3* (Nco1+BglII)	*LEU2 URA3*	[20]
pAS1008	pAS924	HFStag-*SMT3* (NcoI+BglII)	*LEU2 URA3*	This study
pYT1014	pAS924	HFGFP-*SMT3* (SacI+BglII)	*LEU2 URA3*	[11]
pYT1033	pTS901IU	*TOP2*∶*HA* (SpeI)	*URA3*	[20]
pYT1035	pTS901IU	*top2*Δ*C*∶*HA* (AvrII)	*URA3*	[20]
pYT1111	pTS901IT	*TOP1*∶*HA* (SalI)	*TRP1*	This study
pYT1131	pTS901IU	*SMC1*∶*HA* (BglII)	*URA3*	This study
pYT1133	pTS901IU	*SMC3*∶*HA* (BglII)	*URA3*	This study
pYT1134	pTS901IU	*SMC4*∶*HA* (BglII)	*URA3*	This study
pYT1135	pTS901IU	*SMC5*∶*HA* (BssHII)	*URA3*	This study
pYT1136	pTS901IU	*SMC6*∶*HA* (HpaI)	*URA3*	This study
pYT1137	pTS901IU	*YCS4*∶*HA* (Bgl II)	*URA3*	This study
pYT1138	pTS901IU	*YCS5*∶*HA* (SpeI)	*URA3*	This study
pYT1146	pTS904CU	*smc2KR6*	*URA3*	This study
pYT1145	pTS901IU	*smc4KR4*	*URA3*	This study
pT-115	pTS911CU	*pGAL*∶*SIZ1*	*URA3*	[62]
pT-203	pTS911CU	*pGAL*∶*SIZ2*	*URA3*	This study
pT-23	pTS910CU	*SIZ1*∶*GFP*	*URA3*	[63]
pT-81	pTS910CU	*siz1Δ440*∶*GFP*	*URA3*	[9]

**Table 2:** Yeast strains.

Strains	Relevant genotype	Source
Isogenic to W303-1A	*MATa ade2-1ura3-1trp1-1leu2-3,112 his3-11,15 can1-100*	R. Rothstein
HF-W303	*MATa HF-SMT3::LEU2*	This study
HFS-W303	*MATa HFStag:SMT3::LEU2*	This study
HFG-W303	*MATa HFGFP:SMT3::LEU2*	This study
1aYT629	*MATa NOP1:mRFP::URA3 HFGFP:SMT3::LEU2 slx8Δ::KanMX*	This study
1cYT630	*MATa URA3 HFGFP:SMT3::LEU2 ubc9-1 (congenic)*	This study
1cYT628	*MATa siz1::c.g.HIS3 siz2::LEU2 mms21-CH::HIS3 pT-23 / pTS910CU-SIZ1*	This study
1YT632	*MATa siz1::c.g.HIS3 siz2::LEU2 mms21-CH::HIS3 pT-81 / pTS910CU-siz1Δ440*	This study
NOP1-1cYT628	*MATa siz1::c.g.HIS3 siz2::LEU2 mms21-CH::HIS3 NOP1:mRFP::URA3 / pTS910CU-SIZ1*	This study
NOP1-1YT632	*MATa siz1::c.g.HIS3 siz2::LEU2 mms21-CH::HIS3 NOP1:mRFP::URA3 / pTS910CU-siz1Δ440*	This study
10aYT633	*MATa siz1::c.g.HIS3 siz2::LEU2 mms21-CH::HIS3 / pTS911CU-SIZ1*	This study
11aYT634	*MATa siz1::c.g.HIS3 siz2::LEU2 mms21-CH::HIS3 / pTS911CU-SIZ2*	This study
1033-W303	*MATa TOP2:HA::URA3*	[11]
1035-W303	*MATa top2ΔC:HA::URA3*	[11]
15cYT636	*MATa TOP2:HA::URA3 mms21-CH::HIS3*	This study
16cYT637	*MATa top2ΔC:HA::URA3 mms21-CH::HIS3*	This study
17-YT635	*MATa mms21-CH::HIS3*	X. Zhao
18-22aYT636	*MATa mms21-CH::HIS3 top1Δ0::kanMX (congenic)*	This study
YT638	*MATa/α TOP1/top1Δ0::kanMX TOP2/top2ΔC:HA::URA3 mms21-CH::HIS3/mms21-CH::HIS3*	This study
EJY457	*MAT* ***a*** * TOP1-K65,91,92R- HA-His_8_::HIS3 [cir°]*	[39]
21cYT639	*MATa TOP1-K65,91,92R- HA-His_8_::HIS3 mms21-CH::HIS3*	This study
22cYT640	*MATa TOP1-K65,91,92R- HA-His_8_::HIS3 mms21-CH::HIS3 top2ΔC:HA::URA3*	This study
1131-W303	MAT@ *SMC1:HA::URA3*	This study
924-1131-W303	*MATa HF-SMT3::LEU2 SMC1:HA::URA3*	This study
1008-1131-W303	*MATa HFStag:SMT3::LEU2::LEU2 SMC1:HA::URA3*	This study
1133-W303	*MATa SMC3:HA::URA3*	This study
924-1133-W303	*MATa HF-SMT3::LEU2 SMC3:HA::URA3*	This study
1008-1133-W303	*MATa HFStag:SMT3::LEU2::LEU2 SMC3:HA::URA3*	This study
1134-W303	*MATa SMC4:HA::URA3*	This study
924-1134-W303	*MATa HF-SMT3::LEU2 SMC4:HA::URA3*	This study
1008-1134-W303	*MATa HFStag:SMT3::LEU2::LEU2 SMC4:HA::URA3*	This study
1135-W303	MAT@ *SMC5:HA::URA3*	This study
924-1135-W303	*MATa HF-SMT3::LEU2 SMC5:HA::URA3*	This study
1008-1135-W303	*MATa HFStag:SMT3::LEU2::LEU2 SMC5:HA::URA3*	This study
1136-W303	*MATa SMC6:HA::URA3*	This study
924-1136-W303	*MATa HF-SMT3::LEU2 SMC6:HA::URA3*	This study
1008-1136-W303	*MATa HFStag:SMT3::LEU2::LEU2 SMC6:HA::URA3*	This study
640-YW0100s	*MATa SMC4:GFP::URA3 SIK1:mRFP::kanMX ubc9-1*	This study
1111-W303	*MATa TOP1:HA::TRP1*	This study
924-1111-W303	*MATa HF-SMT3::LEU2 TOP1:HA::TRP1*	This study
1aYT641	*MATa HF-SMT3::LEU2 TOP1:HA::TRP1 siz1::c.g.HIS3 siz2::LEU2*	This study
1bYT642	*MATa HF-SMT3::LEU2 siz1::c.g.HIS3 siz2::LEU2*	This study
1cYT643	*MATa HF-SMT3::LEU2 mms21-CH::HIS3*	This study
1YT644	*MATa HF-SMT3::LEU2 SMC3:HA::URA3 mms21-CH::HIS3*	This study
1YT645	*MATa HF-SMT3::LEU2 SMC1:HA::URA3 mms21-CH::HIS3*	This study
1YT646	*MATa HF-SMT3::LEU2 SMC4:HA::URA3 mms21-CH::HIS3*	This study
Isogenic to BY4743	*MATa ade2 his3 leu2 lys2 trp1 ura3*	ATCC
1014-YPH499b	*MATa HFGFP:SMT3::LEU2*	This study
1dYT631	*MATa HFGFP:SMT3::LEU2 slx5Δ::KanMX*	This study
1035-BY4729	*MATα top2ΔC:HA::URA3*	[11]
1aYT625	*MATα top2ΔC:HA::URA3 top1Δ0::kanMX*	[11]
1aYT624	*MATα TOP2:HA::URA3 top1Δ0::kanMX*	This study
1146-YT656	*smc2Δ::kanMX / smc2KR6 URA3*	This study
1YT657	*smc4KR4:HA::URA3*	This study
1YT658	*smc4KR4:HA::URA3 smc2Δ::kanMX /smc2KR6::URA3*	This study
YT659	*MATa/MATα mms21-CH TOP1/top1Δ TOP2/top2-SNM::kanMX*	This study
YT660	*MATa/MATα SMC2/smc2KR6::URA3 top1Δ/top1Δ TOP2/top2ΔC::LEU2*	This study
Isogenic to YPH499	*MATa ura3-52 lys2-801 ade2-101 trp1Δ63 his3Δ200 leu2Δ1*	Ph.Hieter
924-YPH499b	*MATa HF-SMT3::LEU2*	[20]
1008-YPH499	*MATa HFStag:SMT3::LEU2*	This study
1014-YPH499	*MATa HFGFP:SMT3::LEU2 slx5Δ::KanMX*	This study

In order to replace the wild type *SMT3* gene with the tagged versions HFS-*SMT3* (6xHis, FLAG, S-tag) and HFG-*SMT3* (6xHis, FLAG, GFP), the targeting constructs were generated based on the HF-*SMT3::LEU2* (6xHis, FLAG-tagged SUMO) integrative plasmid pAS924 [20]. The PCR-generated fragments of S-tag and GFP were inserted into the SpeI site between the *SMT3* promoter and ORF in pAS924. The S-tag sequence had an addition of the Protein A gene fragment (as a stuffer) from the modified TAP-tagging vector [59]. To generate the *SMT3* replacing fragments, the resulting plasmids pAS1008 (HFS-*SMT3*) and pYT1014 (HFG-*SMT3*) were digested with NcoI/BglII and SacI/BglII, respectively.

To purify His-tagged SUMO conjugates from yeast cells carrying pAS924, pAS1008, or pYT1014 *SMT3* gene replacements, 50-ml cultures were harvested, cells were disrupted by glass beads (15 min) in 500 µl lysis buffer (0.1 M Tris pH 8.0, 6 M guanidine chloride, 0.5 N NaCl, 10 mM N-ethylmaleimide, NEM), and extracts were clarified by centrifugation. The clarified protein extracts were incubated with nickel-charged Superflow NTA resin (QIAGEN) for 4 hrs. Incubated resin was washed once with the lysis buffer and then three times with the washing buffer (25 mM Tris pH 8.0, 0.3 M NaCl, 0.1% NP-40, 10 mM NEM). Bound protein was eluted by boiling in 1× Laemmli sample buffer. Conjugates and free SUMO were detected by anti-FLAG M2 antibodies (Sigma) after separation by polyacrylamide gel electrophoresis (PAGE).

5xHA (hemagglutinin) tagging of Top1p, Smc1p, Smc2p, Smc3p, Smc4p, Smc5p, and Smc6p was done by cloning the PCR-generated fragments of the corresponding ORFs into the integrative vector pTS901IU [60]. Plasmids can be linearized for integration/replacement using a unique restriction site in the inserted fragment. Details of construction are available upon request. ChIP/qPCR analysis was as described [45].

Fluorescent microscopic imaging was performed on the Zeiss AxioVert microscope equipped with epifluorescence. Z-stacks of 20 images were taken at 0.2 µM intervals.

## Supporting Information

Figure S1 Genetic interaction between *top1Δ*,*top2-SNM* and mms21-CH mutations. (Left panel) A sample of tetrad analysis for the diploid strain (YT659) homozygous for *mms21-CH* and heterozygous for *top2-SNM* and *top1Δ* Arrows point to triple mutant clones. (Right panel) Surviving *mms21-CH top2-SNM top1Δ* triple mutants have synthetic growth defect. Two independent viable YT659 spores are shown for the triple mutant and for the double *mms21-CH top2-SNM* mutant.
(0.47 MB PDF)

Figure S2 Non-SMC subunits of condensin are sumoylated in mitosis. SUMO conjugates were purified by IMAC from YCS5∶5HA (1138-W303, 924-1138-W303) and YCS4∶5HA (1137-W303, 924-1137-W303). Total SUMO conjugates were detected by anti-FLAG (M2) antibody. The arrows indicate the free form of tagged SUMO.
(0.20 MB PDF)

Figure S3 Sumoylation levels of Smc2p and Smc4 in mitosis are decreased in SUMO acceptor lysine mutants. SUMO conjugates were purified by IMAC from *smc2KR6* (1146-YT656) and *smc4KR4* (1YT657) and corresponding wild type control strains. Total SUMO conjugates were detected by anti-Smt3p antibody (Abcam). The arrows indicate the free form of tagged SUMO.
(0.37 MB PDF)
