# Supplementary figures and images for: Cooperation of Sumoylated Chromosomal Proteins in rDNA Maintenance
Source: PLoS Genet. 2008 Oct 10;4(10):e1000215. doi: 10.1371/journal.pgen.1000215 (PMC2563031; doi:10.1371/journal.pgen.1000215)

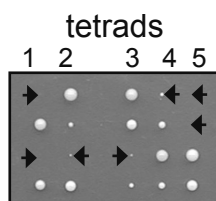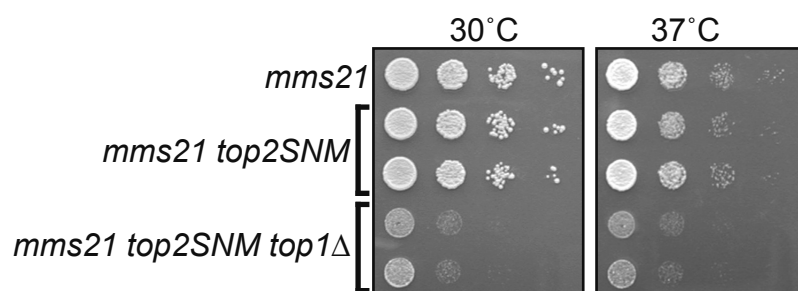

Supplement: Figure S1 — Genetic interaction between top1Δ,top2-SNM and mms21-CH mutations. (Left panel) A sample of tetrad analysis for the diploid strain (YT659) homozygous for mms21-CH and heterozygous for top2-SNM and top1Δ Arrows point to triple mutant clones. (Right panel) Surviving mms21-CH top2-SNM top1Δ triple mutants have synthetic growth defect. Two independent viable YT659 spores are shown for the triple mutant and for the double mms21-CH top2-SNM mutant. (0.47 MB PDF) [file pgen.1000215.s001.pdf]

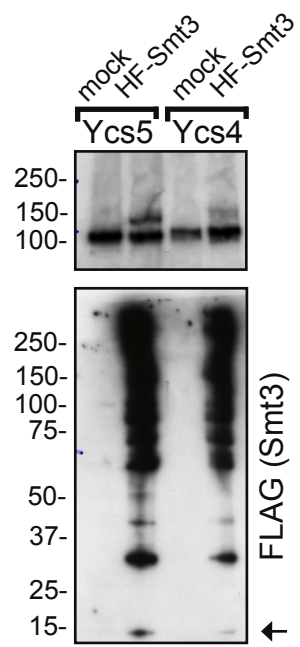

Supplement: Figure S2 — Non-SMC subunits of condensin are sumoylated in mitosis. SUMO conjugates were purified by IMAC from YCS5∶5HA (1138-W303, 924-1138-W303) and YCS4∶5HA (1137-W303, 924-1137-W303). Total SUMO conjugates were detected by anti-FLAG (M2) antibody. The arrows indicate the free form of tagged SUMO. (0.20 MB PDF) [file pgen.1000215.s002.pdf]

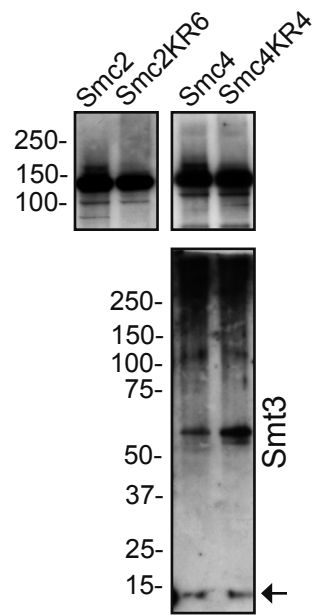

Supplement: Figure S3 — Sumoylation levels of Smc2p and Smc4 in mitosis are decreased in SUMO acceptor lysine mutants. SUMO conjugates were purified by IMAC from smc2KR6 (1146-YT656) and smc4KR4 (1YT657) and corresponding wild type control strains. Total SUMO conjugates were detected by anti-Smt3p antibody (Abcam). The arrows indicate the free form of tagged SUMO. (0.37 MB PDF) [file pgen.1000215.s003.pdf]
